# Protein Complexes in Urine Interfere with Extracellular Vesicle Biomarker Studies

**DOI:** 10.5772/62579

**Published:** 2016-03-01

**Authors:** Magda Wachalska, Danijela Koppers-Lalic, Monique van Eijndhoven, Michiel Pegtel, Albert A. Geldof, Andrea D. Lipinska, R. Jeroen van Moorselaar, Irene V. Bijnsdorp

**Affiliations:** 1 Department of Urology, VU University Medical Center, Amsterdam, The Netherlands; 2 Department of Virus Molecular Biology, Intercollegiate Faculty of Biotechnology UG-MUG, University of Gdańsk, Gdańsk, Poland; 3 Department of Pathology, VU University Medical Center, Amsterdam, The Netherlands; 4 Department of Neurosurgery, VU University Medical Center, Amsterdam, The Netherlands

**Keywords:** Urine Extracellular Vesicles, Ultracentrifugation, Tamm-Horsfall Protein, Protein-complex, MicroRNA, Size-exclusion Chromatography

## Abstract

Urine exosomes (extracellular vesicles; EVs) contain (micro)RNA (miRNA) and protein biomarkers that are useful for the non-invasive diagnosis of various urological diseases. However, the urinary Tamm-Horsfall protein (THP) complex, which forms at reduced temperatures, may affect EV isolation and may also lead to contamination by other molecules including microRNAs (miRNAs). Therefore, we compared the levels of three miRNAs within the purified EV fraction and THP- protein-network. Urine was collected from healthy donors and EVs were isolated by ultracentrifugation (UC), two commercial kits or sepharose size-exclusion chromatography (SEC). SEC enables the separation of EVs from protein-complexes in urine. After UC, the isolation of EV-miRNA was compared with two commercial kits. The EV isolation efficiency was evaluated by measuring the EV protein markers, Alix and TSG101, CD63 by Western blotting, or miR-375, miR-204 and miR-21 by RT-qPCR. By using commercial kits, EV isolation resulted in either low yields or dissimilar miRNA levels. Via SEC, the EVs were separated from the protein-complex fraction. Importantly, a different ratio was observed between the three miRNAs in the protein fraction compared to the EV fraction. Thus, protein-complexes within urine may influence EV-biomarker studies. Therefore, the characterization of the isolated EV fraction is important to obtain reproducible results.

## 1. Introduction

Extracellular vesicles (EVs) are small spherical structures (30–200 nm) that are composed of a lipid bilayer. Different classes of EVs have been assigned, and definitions and proposed nomenclature, like exosomes, microvesicles and apoptotic bodies, have been discussed [1–3]. EVs are actively released from cells and contain proteins and nucleic acids that partly reflect the cellular content [4]. EVs are involved in intercellular signalling [5,6] and can be found in biofluids such as urine [7]. As biofluids are easily accessible, EVs have gained wide interest as a source of biomarkers for diagnostic or prognostic measures [8]. Urine EVs are derived from epithelial cells lining within the urinary tract [9] and are very stable [10]. EV isolation methods are increasingly becoming available, providing faster, easier and less labour intensive techniques for EV isolation. However, these commercially available kits all need to be evaluated and isolated fractions need to be characterized.

For isolating urine EVs, the presence of the Tam Horsfall protein (THP; uromodulin), which forms polymers at lower temperatures, may decrease the EV yield. THP has been described to trap EVs. Therefore, EVs may pellet at a lower speed centrifugation [11]. However, it has not yet been studied to what extent the THP-protein complex binds free miRNAs, and how this contributes to the levels of detected miRNAs within EVs. Therefore, we investigated to what extent THP polymers contribute to the expression of miRNA within EVs. Using size-exclusion chromatography (SEC), we separated the urine protein complexes from the EV fraction. Interestingly, the protein complexes contained miRNAs and, of the three tested miRNAs, we found a different relationship compared to that within the EVs, indicative of a different biomarker profile. Therefore, when using any type of urine EV isolation method, the contribution of protein-complexes when performing biomarker identification studies should be carefully considered.

## 2. Methods

### 2.1 Urine EV Isolation

Urine was collected from healthy individuals and stored at −80°C. The urine EVs were isolated by differential (ultra)centrifugation (UC), as previously described [5]. To break down the THP-polymers, dithiothreitol (200 mg/ml; DTT) was added where indicated for 1 h at 37°C to the THP pellet (15,000 x g pellet), and the supernatant was used for EV isolation by UC as, previously described [12]. For a comparative analysis, both Norgen Biotek urine EV RNA isolation kit (#47200, Norgen Biotek, Thorold, ON, Canada) and Life Technologies EV isolation kit (# 4484452 Life Technologies, Mulgrave, VIC, Australia) were used, according to the manufacturer's instructions. The ultrastructural evaluation of the urine EVs was performed by a transmission electron microscopy (TEM), as previously described [4].

### 2.2 The miRNA Expression Levels

RNA was isolated by Trizol, as previously described [4]. The miRNA expression was determined by RT-PCR, according to the manufacturer's instructions and as previously described [4]. We selected miR-21, miR-204 and miR-375 (Life Technologies, Grand Island, MY, USA; miR-21 #000397, miR-204 #000508 and miR-375 #000564), which were described to be present in the urine EVs.

### 2.3 Protein Analyses

Western blot was performed, as previously described [5]. For the measurement of CD63 protein levels, non-reduced conditions were used. The membranes were incubated with mouse anti-Alix (1:500; Cell Signaling), goat anti-TSG101 (1:1000; Santa Cruz Biotechnology, USA), rabbit anti-THP (1:1000; Santa Cruz Biotechnology) or mouse anti-CD63-antibody (1:1000; Santa Cruz Biotechnology). The overall protein yield was determined by Coomassie Brilliant Blue (R-250, Merck, France) staining on the total gels, as previously described [5].

### 2.4 Size Exclusion Chromatography

SEC was performed according to the protocol described by Böing et al. [13], with small modifications. In brief, sepharose (CL-4B/2B 30 mL, GE Healthcare; Uppsala, Sweden) was washed with PBS containing 0.32% trisodiumcitrate (pH 7.4, 0.2 μm filtered). Subsequently, the tip of a 10 mL plastic syringe (Becton Dickinson (BD), San Jose, CA) was stuffed with a nylon stocking (20 denier, Hema, Amsterdam, the Netherlands). The syringe was stacked with 10 mL washed sepharose to create a column with a diameter of 1.6 cm and height of 6.2 cm. The urine was centrifuged 500 x g 15 minutes and 1.5 mL of it was loaded on the column, followed by elution with PBS/0.32% citrate (pH 7.4, 0.02 μm filtered). The eluate was collected in 26 sequential fractions of 0.5 mL. Each fraction was stored at −80°C until further processing.

## 3. Results

### 3.1 Ultracentrifugation and Commercial EV Isolation Kits

**Figure 1.**
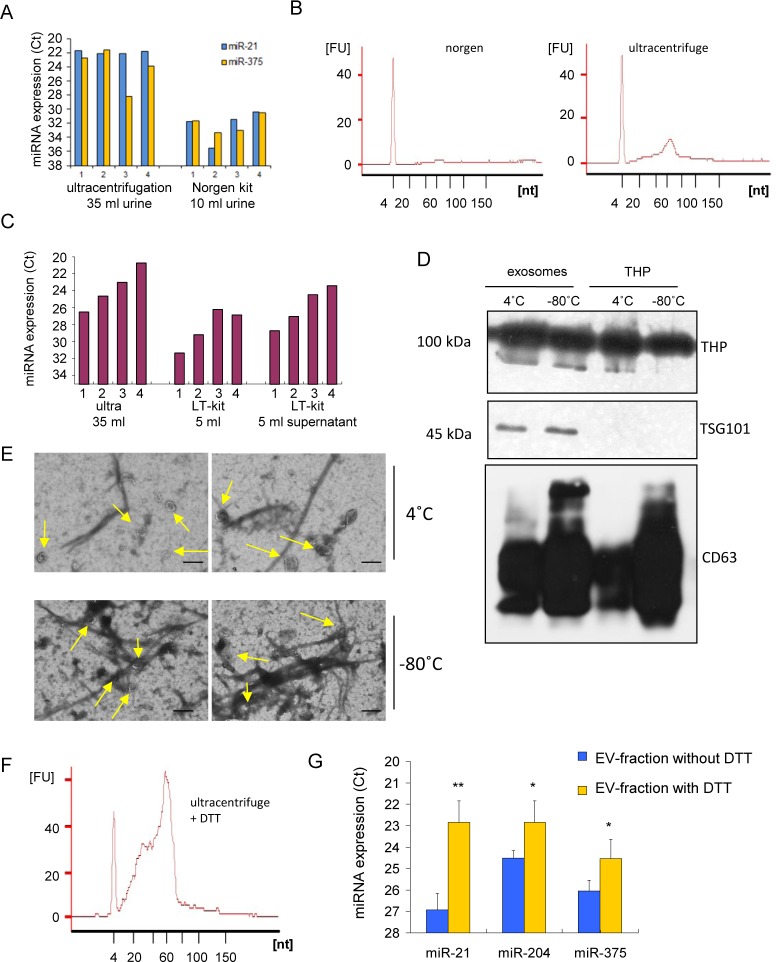
**Urine extracellular vesicle isolation methods**. A. Expression of miR-21 and miR-375 in urine extracellular vesicles, comparing ultracentrifugation with Norgen kit. B. Small RNA profile of isolated extracellular vesicles after Norgen and ultracentrifugation. C. Expression of miR-21 in urine of four different donors, comparing ultracentrifugation (ultra) to that of the Life Technologies kit (LT) and the supernatant (SN). D. Western blot showing the expression of THP, TSG101 and CD63 in extracellular vesicles and THP fraction. E. EM pictures showing extracellular vesicles trapped within the THP-network, after storage of 4C or −80C. Size bar = 200 nm. F. The effect of the addition of 200 mg/ml DTT to the THP-pellet on the smallRNA profile before extracellular vesicles isolation by ultracentrifugation. G. miR-21, miR-204 and miR-375 expression levels after releasing extracellular vesicles from the THP network by the addition of DTT, showing an increase in the expression levels. Each value represents the mean ^+^/_-_ SD of three donors. ** p<0,01, *p<0,05.

As UC is very labour intensive, we determined whether commercial EV isolation kits are easier to use improved the EV yield. To determine the EV isolation yields using the commercial Norgen kit and Life Technologies (LT)-kit, we compared the expression of miR-21 and miR-375. EV isolation using both kits was considered to be very user friendly, fast and easy. For both kits, we slightly modified the protocol to remove debris from the samples. We then added one additional 15,000 x g centrifugation step, as commonly used for UC protocols. However, after using the Norgen kit (10 ml urine), both of the tested miRNAs were below the detection limit (Figure 1A). The output of this kit is a purified RNA fraction, though hardly any small RNAs were detected. This indicates that the isolation efficacy was very poor (Figure 1B). EVs isolation by the LT-kit resulted in higher miRNA levels (Figure 1C) by using only 5 ml of urine as a starting material. Increasing the volume of urine, while not increasing the solution given by LT, did not result in an increase in the levels of the tested miRNAs. This is probably related to the maximum binding capacity obtained with 5 ml urine. The miRNA levels in the EVs after UC (in which sevenfold more urine was used) were >20 times higher compared to the LT-kit (Figure 1C). When the EVs were isolated from the unbound fraction from the LT-kit (supernatant) by UC, we measured the high miRNA levels that seemed to be in relation with the total miRNA levels that were present in the sample.

### 3.2 THP Traps EVs

To determine the contribution of protein complexes to the RNA signal, we determined the level of EV-capture within the THP-network. Storage at reduced temperatures activates the THP-complex formation. We determined the effect of THP on EV isolation and compared the equal input volumes before isolation as normalization (Figure 1D). THP was found at comparable levels when the urine was kept at either 4°C or −80°C (Figure 1D). The EV marker, CD63, was detected in both the EV (UC) and THP-pellet fraction (15,000 x g fraction), indicating that EVs may, indeed, be present in both fractions (Figure ID). However, the EV marker, TSG101, was not detected in the THP-fraction, which indicates that the EV number in this fraction was low (Figure 1D). To determine the amount of EVs that were captured in the THP network, we performed an electron microscopy. A small fraction of EVs were trapped in the THP-network (Figure 1E). The number of EVs in the THP pellet was slightly increased after storage at −80°C, as well as the presence of THP-complexes, compared to urine storage at 4°C, indicating that THP reduced EV isolation to a small extent (Figure 1E). After breaking down the THP network by incubation with DTT, small RNA levels were highly increased in the EV fraction obtained after UC (Figure 1F), compared to normal ultracentrifugation only (Figure 1B). The levels of miR-375, miR-204 and miR-21 in the EVs isolated with UC, combined with DTT-breakdown fraction, was increased by ∼10 times (Figure 1G).

3.3 *Size Exclusion Chromatography*

**Figure 2.**
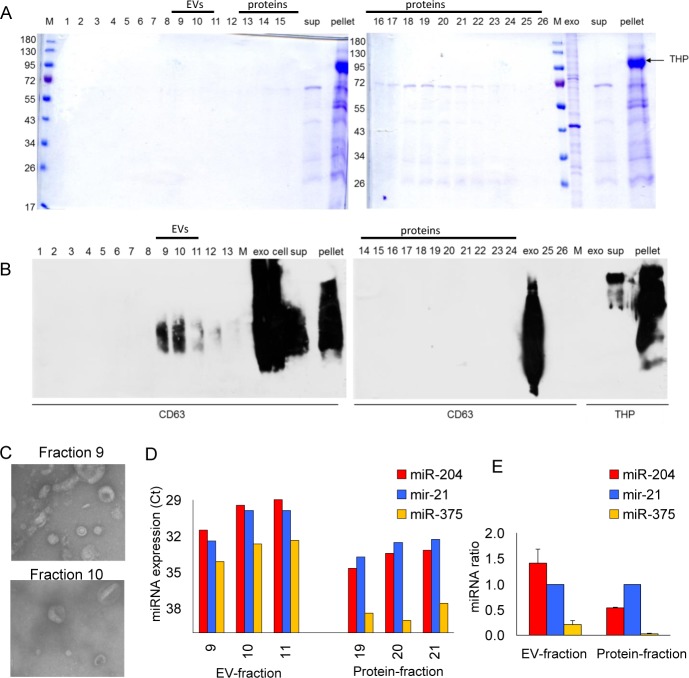
**Contribution of proteins to extracellular vesicle isolations**. A. Coomassie staining of the lysate of 26 fractions from size exclusion, compared to the supernatant, purified extracellular vesicle (EVs isolated by ultracentrifugation) and THP-pellet. B. Western blot showing CD63 levels in each of the 26 fractions from size exclusion, compared to the supernatant, purified extracellular vesicles (ultracentrifugation) and THP-pellet. C. Electron microscopy pictures of extracellular vesicles isolated within fractions 9 and 10. D. Expression of miR-204, miR-21 and miR-375 in extracellular vesicles fractions 9-11 and protein fractions 19–21. E. Changed ratio between miR-204 and miR-375 related to miR-21, indicating that the protein fraction contains a different subset of miRNAs than the extracellular fraction.

Besides EVs, protein-complexes are released by a breakdown of the THP-polymer network after adding DTT. By single-step SEC, EVs can be separated from the protein complexes, with urine as the starting material and without adding denaturating chemicals to the samples [13]. Within serum, it has been described that EVs are present in fractions nine and 10 and proteins in fractions 16–32 [13]. In the fractions 16–23, we observed proteins, which is in agreement with the observations of Nieuwland et al. [13] (Figure 2A). The exosomal marker, CD63, was observed in fractions nine and 10, and slightly in fractions 11–13 (Figure 2B). By EM, we confirmed the presence of 100 nm sized EVs in fraction 9 and 10 (Figure 2C), similar to observations in human serum [13] and plasma (Pegtel et al., unpublished data). To determine the miRNA expression levels and ratio, we performed RT-qPCR and detected miRNAs within the EVs, and the ratio between fractions nine, 10 and 11 were comparable (Figure 2D). On the other hand, in the protein-fractions, the ratio between the measured miRNAs was different compared to the EV fraction (Figure 2D-E). This indicates that, in urine, miRNA containing protein complexes may contain a distinctive set of biomarkers compared to those found within EVs.

## 4. Discussion

The results from the present study demonstrate that polymeric-THP, which is formed at reduced temperatures in urine, traps EVs and contains miRNAs. These protein-complexes have a different miRNA ratio of miR-21, miR-375 and miR-204 compared to the EV fraction. Therefore, when using urine EVs as a source for biomarker identification studies, both commercial EV isolation methods and adding reducing agents, such as DTT, should be carefully checked for contamination with protein-bound RNA.

EVs are secreted vesicles that are considered potential sources for biomarkers. Urinary EVs isolation usually contains a two-step differential centrifugation process. During an initial 15,000xg run, high-density membranes and THP polymers are removed. After the second step at 100,000×g, urinary EVs are normally found in the pellet [15]. Previously, it was demonstrated that storage at −20°C dramatically decreased the number of EVs [10], while long-term storage at −80°C or short-term storage at 4°C resulted in comparable EV yield [10,12], which is in agreement with our observations. The two tested commercial kits provided a more easy and rapid method. However, repeated testing of the Norgen kit resulted in extreme low RNA yields. Unfortunately, this kit does not allow the characterization of the isolated fraction, as the output is RNA only. The kit of Life Technologies was extremely easy and fast, though isolation of EVs was inefficient. Furthermore, both of the isolation kits make use of a protocol that does not include a high-speed pre-centrifugation step (15,000 x g). Therefore, protein complexes are potentially also captured using the kits tested in this study.

The presence of THP in urine may reduce EV isolation yields. THP is a major protein component of urine [16]. THP polymer had a rope-like structure, in agreement with the observation of others [12,17]. Polymeric THP has been described to entrap large amounts of EVs, which may pellet at high speed [14], reducing EV yields. DTT can release EVs from the THP polymeric network [10,12]. However, monomeric THP will be present in the EV fraction [12,18,19], potentially interfering with proteomic and RNA-assays. We observed EVs in the THP-pellet, though, compared to the total EV fraction, the number was relatively limited. It is most likely that miRNAs are trapped by THP network, which contribute to the higher biomarker signals observed in previous studies, compared to the content in EVs. By destroying this network with a reducing agent, EVs are released, as well as protein-complexes that contain (micro)RNA. Therefore, THP may interfere with measuring the expression of markers when changing EV isolation methods. In conclusion, urine EVs are a potential source of the identification of biomarkers. Storage of urine and further processing may affect the biomarker profile due to the contribution of protein-complexes that also contain RNA. We show that the contribution of protein-complexes in urine, such as THP, requires detailed characterization before proceeding with RNA or protein-based marker profiles for disease detection.
